# How the COVID-19 Pandemic Affected the Functioning of Tourist Short-Term Rental Platforms (Airbnb and Vrbo) in Polish Cities

**DOI:** 10.3390/ijerph19148730

**Published:** 2022-07-18

**Authors:** Joanna Kowalczyk-Anioł, Karolina Kacprzak, Ewa Szafrańska

**Affiliations:** 1 Institute of Urban Geography, Tourism Studies and Geoinformation, Faculty of Geographical Sciences, University of Lodz, 90-136 Łódź, Poland; joanna.kowalczyk@geo.uni.lodz.pl; 2 CiTUR Center for Tourism Research, Development and Innovation, Polytechnic of Leiria, 2411-901 Leiria, Portugal; 3 Doctoral School of Social Sciences, University of Lodz, 90-136 Łódź, Poland; karolina.kacprzak2@edu.uni.lodz.pl

**Keywords:** COVID-19, tourism, short term rental (STR), Airbnb, Vrbo, Polish cities

## Abstract

The article presents the impact of the COVID-19 pandemic on urban tourism activity on short-term rental (STR) platforms in Central and Eastern Europe (CEE). It offers empirical evidence of how Airbnb and HomeAway (Vrbo) changed in Polish cities during the COVID-19 pandemic. A case study of Polish cities was also used to investigate what pandemic-induced scenarios of that impact are presented in the literature. In particular, the study identifies “loser” cities, in which the pandemic consolidated and deepened the decline in active STR volume, and “winner” cities, in which the volume and dynamics of the active STRs increased during the pandemic.

## 1. Introduction

Before the pandemic, 21st century cities were frequently developing towards the model of the lively city that is attractive to residents and other city users alike. On the eve of the pandemic, many cities were even competing for tourists and residents on the basis of their being lively cities with lively spaces and infrastructure. The lively neighbourhood was the focus of, for example, a new urban tourism that was “off the beaten track” [1,2]. At the same time, lively neighbourhoods became an important and prominent feature of Airbnb’s offer, a 21st century tourist innovation belonging to the so-called sharing economy in tourism (SET) [3]. The active rentals centred around a core of tourist accommodation in the local housing stock provided by residents (peer-to-peer) via online tourist short-term rental (STR) platforms such as Airbnb and HomeAway (further, see [4]).

Meanwhile, STR/Airbnb were touted as an opportunity for residents with surplus housing resources to make additional earnings as hosts and as an alternative to traditional accommodation [5]. This accommodation was to be particularly desirable to tourists looking for authentic “local” experiences (living like locals) [6,7,8] (It is worth adding that some tourists develop such strong ties with an attractive tourist destination that they become repeat medium-term renters or buy real estate to “live like locals”, as shown in an interesting way by, among others, Attansi et al. (2013, 2019) [9,10]). For some destinations it presented a solution to an underdeveloped accommodation base [11,12]. However, in the second decade of the 21st century, in mass tourism cities, where STRs were recognised as a new business opportunity, there was a clear professionalisation of hosting and a commercialisation of Airbnb (STR) business [13,14,15]. The changes observed were, according to many, contradictory to the basic principles of the sharing economy [16]. The so-called “platform economy” [17] fuelled tourism, the financialization of housing and gentrification, including in previously non-touristic neighbourhoods (e.g., [18,19]). Prior to the pandemic, the rapid proliferation of STRs in cities began to feature more heavily in the discussion of overtourism, including in Polish cities (e.g., [20,21]).

Local stakeholders’ perceptions of the negative effects of global homesharing platforms in cities resulted in numerous calls for legal regulation—and even restrictions—on the operation of STR platforms [22,23]. There was a focus on, among other things, the unhealthy growth in the proportion of “entire apartments” among the active rentals on homesharing platforms, which accompanied the financialisation of housing and a strengthening of the platform economy. These changes led to the exploitation of ordinary but lively and diverse neighbourhoods [24] and the displacement of their inhabitants [25].

On the other hand, an analysis of the EU’s long-term position on proposals and measures by urban authorities to regulate the short-term rental market at the EU level [26] shows that the sharing economy is still seen as a promising sector of the European economy that focuses on better use of resources. European Union authorities have not introduced common regulations thus far, and the acts they issue contain only guidelines for the regulation of short-term rentals in Member States [26]. Significant restrictions on the functioning of STRs have been introduced in Germany and France, and lesser ones in Portugal [26]. However, specific forms of legislative STR regulations have been introduced locally in many European cities [22,27,28]. In Poland, such attempts are under discussion [26,29].

The COVID-19 pandemic brought a great threat to public health [30], side-effects on the EU economy as a whole [31,32,33,34] and challenges to the organisation of life in cities [35,36,37,38]. COVID-19—as a dual health and economic crisis—has also changed the way of thinking about housing, in particular about the long-term rental market [39]. The initial phases in particular, which involved radical restrictions on mobility and lockdowns significantly changed the determinants of tourism in cities [3,40]. In many tourism cities, pandemic limitations had major repercussions for the economy [41,42]. Actions undertaken at local, regional and national levels supported the urban tourism sector to varying degrees [3,43,44]. The case was slightly different for STRs/Airbnb. In most cities (incl. in Poland), a significant proportion of tourist apartments and STR apartments were not officially registered, remaining “invisible” [45]. Thus, the activities of many Airbnb “hosts” had no access to support measures (e.g., financial compensation for the period of the mandatory ban on activities or for lost potential profits).

At the same time, the restrictions introduced on tourist mobility, or bans on tourism industry operations (incl. tourist rental via online platforms [46]) and the general decline in interest in urban tourism seen during the pandemic (and anticipated for the post-pandemic period) [47] mean that we are now asking how the pandemic actually affected the functioning of STR platforms (such as Airbnb) in cities. The literature provides no clear-cut answer. Some have suggested that tourists wanting to avoid crowded spaces would more readily choose STRs over traditional accommodation options [48]. Others, conversely, predicted that the pandemic would significantly reduce STRs (e.g., [49]). Meanwhile, Medeiros et al. (2022) indicate that, compared to the hospitality industry, Airbnb is more resilient to the potential external shocks that threats (incl. economic crises and natural disasters) might bring to demand and/or supply [50].

In view of the above, the article first identifies the main scenarios for Airbnb’s development during the pandemic that are presented in the literature; then, it uses an analysis of data provided by AirDNA (https://www.airdna.co) to show changes in the number and breakdown of active Airbnb (and Vrbo) rentals in selected cities in Poland during the pandemic. We conclude with a summary in which we discuss the results and compare them against the identified scenarios.

The article is documentary, and its main source material is data from the AirDNA portal, which Pawlicz and Prentice (2021) and Adamiak (2022) identify as the most popular commercial provider of data about the STR market that are usually not included in official statistics [45,51]. Thus, the article fills the research gap in identifying changes in the activities of STRs in Central and Eastern Europe cities (on the example of Polish cities) during the pandemic.

Since it was established in 2008, Airbnb has grown to become the largest STR platform in the world (5.6 million active apartment and room rentals in 2021 [45], and, after going public in December of 2020, it overtook Booking Holdings to become the world’s most valuable tourism company, with capital exceeding USD 100 billion [45]. The Vrbo platform was established in 1995 as one of the pioneers of online tourist rental. Its acquisition by Expedia and merger with HomeAway saw it offering over two million flats and apartments [45]. Bearing this in mind, and the fact that Airbnb is commonly recognised in the literature as the epitome of the platform economy in tourism [14], the literature review focuses on this platform. Due to the nature of the data available from AirDNA, the data analysis also includes Vrbo (which as of March 2022 had a share that ranged in various cities from 1% in Zakopane, Szczecin, Łódź, Katowice, Toruń to 20% in Świnoujście). The only city with no active rentals on the Vrbo platform is Lublin.

Geographically, the article focuses on the Polish cities with the most accommodation offers on Airbnb and Vrbo (in the 3rd quarter of 2019). These are Kraków, Warsaw, Gdańsk, Wrocław, Sopot, Zakopane, Gdynia, Poznań, Szczecin, Łódź, Katowice, Toruń, Lublin and Świnoujście. In Poland, Airbnb appeared in 2012, accompanying the 2012 European Football Championships (UEFA Euro 2012) [52], and currently shares the position of most popular platform in Poland with Booking.com [45]. Up until the pandemic, Airbnb in Poland had principally focused on major cities, and particularly on recognised tourist centres [45,52,53]. Entire homes constituted a considerably larger proportion of the accommodation on offer on the platform than did shared apartments (i.e., private or shared rooms), and there was a clear trend towards the professionalisation of hosts and active rentals on the platform [11,52,54,55]. To date, there has not been a separate analysis of Vrbo in the Polish literature. However, the latest works on the pandemic based on AirDNA (e.g., [56]) looking at housing market dynamics in Warsaw have included the platform (see also [45]). On the eve of the pandemic, i.e., in September of 2019, the symptoms of saturation appeared in the tourist apartment markets of five tourist cities—Warsaw, Kraków, Gdańsk, Sopot and Wrocław. There was a drop in average occupancy and daily rental rates, and some apartments began to migrate from the short-term model back onto the long-term rentals market [57].

Summarising the above considerations, the research objective is to answer two questions: Have there been any changes in the number of STR facilities in Polish cities due to the COVID-19 pandemic? Have there been any major changes in the breakdown of rental facilities, and in what direction (entire vs. private vs. shared rooms)? The answers to these questions can be used to empirically verify some scenarios presented in the literature regarding the impact of the COVID-19 pandemic on the functioning of STR platforms in cities.

This study contributes to the literature by presenting empirical evidence of the impact of the COVID-19 pandemic on tourism homesharing platforms in urban CEE environments. The case study of Polish cities was also used to verify pandemic-induced scenarios of the impact on STRs presented in the literature. It indicates loser cities, but also winners, where the number and dynamics of active STRs increased during the pandemic.

## 2. Literature Review

The literature review focuses on scientific articles published between the beginning of the COVID-19 pandemic and September 2021. The analysis was conducted in three stages. First was a query of the following Internet databases: ScienceDirect, Scopus and Web of Science. The choice of such multi-search engines was not accidental; it was assumed—after Falagas et al. (2008), Hall (2010) and Romero-García et al. (2019)—that the articles they include significantly shape contemporary scientific discussion [58,59,60] (see also [61]). The purpose of the query was to identify scientific articles relating to the impact of the pandemic on the operation of Airbnb in cities. Three searches were performed on each of the three search engines. The three combinations of keywords were: Airbnb AND COVID-19; Airbnb AND pandemic; Airbnb AND coronavirus. The articles that contained these combinations in their title, abstract or keywords were selected. There were a total of 94 hits in Scopus, 15 in ScienceDirect, and 42 in Web of Science. Duplicate articles were removed from the database, leaving 57 articles. In the third stage, the content of each article was analysed and those that did not address our research topic were discarded. Ultimately, 28 articles qualified for the analysis.

### 2.1. Airbnb (and STRs) during the Pandemic

The COVID-19 pandemic that began in China in late 2019 radically impacted tourism and the operation of homesharing platforms, including Airbnb [62,63,64]. Depending on the local epidemic situation, renting via the platform was limited or sometimes even prohibited [46,62,65]. Local restrictions and restrictions on travel and movement caused changes in booking numbers [41,64,66,67,68] and a drop in foreign visitors [41,67,69] and thus a sharp fall in demand for tourist accommodation [23,62,64,70,71].

The lack of possibilities for rental contributed to huge financial losses for the platform due to, for example, the cancellation or loss of reservations, revenues from additional fees and services [46,67,72] but also to losses for hosts due to loss of income and the need to drop rental prices [46,72,73,74]. In May 2020, the company laid off approximately 1900 employees, i.e., 25% of the entire workforce [23,67].

While predicting the long-term effects of COVID-19 on the STR sector, including Airbnb, is difficult, many academics undertook the task relatively quickly. As a consequence, many studies have already presented initial short-term results (e.g., [64,75]) and proposed possible scenarios and hypotheses regarding the future of the entire sector and/or Airbnb (e.g., [49,76]).

The COVID-19 pandemic forced changes in the STR market. One of the main threats was the risk of contracting the virus [77] and the risk associated with the introduction of lockdowns [78], which affected the breakdown of rented properties. Hu, Lee (2020) and Lim et al. (2020) indicate that tourists, fearing infection, more often cancelled room reservations than entire properties (after: [13]). Similar conclusions were reached by Bresciani et al. (2021) and Benítez-Aurioles (2021), who predicted a drop in the popularity of shared flats. During the first wave of the pandemic in May of 2020, Airbnb managers were obligated to introduce and comply with a special hygiene and cleaning protocol to be able return to the rental market. New rules for landlords were adopted, e.g., the use of personal protective equipment (masks, gloves, disinfectants) or the observation of a 24 h waiting period for entering a facility [79]. Therefore, one of the first consequences of the pandemic was an improvement in OHS standards in homesharing units (e.g., [46,47,80,81,82]).

Some [49,80,83] conclude that the scale of Airbnb’s operation is unlikely to return to pre-pandemic levels. On the other hand, however, Braje et al. (2022) believe that tourists will still be more likely to choose STR (incl. Airbnb) than traditional accommodation facilities, due to, for example, an increase in shorter trips, the delaying of travel due to uncertainty related to the pandemic, and the desire to avoid crowded destinations [13]. Similar conclusions have already been reached by, *inter alia*, Boros and Kovalcsik (2021), Liang et al. (2021) and Gerwe (2021), who believe that accommodation outside the city centre itself will increase, with more peripheral areas being favoured—especially ones with more access to nature [84] or in rural areas [49,84].

Short-term rental service managers are constantly trying to respond to the pandemic by introducing innovative new solutions. For example, the Airbnb 2021 Winter Release was introduced in late 2021 with over 50 improvements making it “easier to host and support the changing needs of travelers”. The most popular new features include: AirCover (Top-to-bottom protection for every Host), Translation Engine (The most advanced translation technology), I’m (even more) Flexible (Searching for more unique homes), Verified Wifi (new speed test tool), Smarter Trips Tab (A redesigned Trips tab with all must-have travel details), Ask a Superhost Expansion (Connects new Hosts to a Superhost for help) [85]. Additionally, Airbnb is trying to acquire new user groups—the disability community (a special Accessibility Review function has been developed) and people travelling with pets. In January 2022, Brian Chesky (co-founder and CEO of Airbnb) posted a short question on his Twitter profile: “If Airbnb could launch anything in 2022, what would it be?” This crowdsourcing garnered him 4000 responses and suggestions. He then shared the top six crowdsourcing ideas, including: crypto payments (top suggestion), clear pricing displays, a guest loyalty programme, updated cleaning fees, more long-term stays and discounts, and better customer service [86]. It can be expected that some of these will soon be included in the Airbnb offer.

### 2.2. Scenarios for the Future of Airbnb and STR

It is clear that the full effect of the coronavirus on STRs will not be visible until some time after the pandemic ends. Forecasting the future of the short-term rental market is difficult, as is assessing whether the changes seen in the homesharing phenomenon and of individual platforms are only temporary [83]. However, a review of the literature shows that much of the attention given to STR (incl. Airbnb) is devoted to scenarios for the future of these platforms after the end and/or slowdown of the pandemic. There is a general perception of pandemic-induced changes in STRs, though the scenarios for potential changes are diverse (Table 1). The analyses differ in perspective—there are supply-and-demand approaches and the voices of housing researchers, tourism researchers and urban researchers. The topic is thus addressed in the pages of journals from various disciplines. In the following part, in line with the aim of the article, the focus is on scenarios of potential changes in STRs, especially those relating to cities.

Some researchers consider STRs/Airbnb in relation to the real-estate market and forecast a transition/outflow of many STRs facilities to the long-term rental sector (scenario 2) [49,64,66,74,83,84,87]. In some cities (e.g., Hong Kong, Warsaw) this phenomenon was already documented in the first year of the pandemic (cf. [56,62,64,68,71,74]). Although shared flats account for a small percentage of Airbnb’s offer, Bresciani et al. (2021) and Benítez-Aurioles (2021) believe that the number of such active rentals will decline (scenario 3). They indicate that, having experienced the pandemic, visitors are more likely to avoid shared housing [47,70]. For sanitary safety reasons, they prefer to rent entire, independent housing units (entire homes/apartments). Some researchers propose quite optimistic scenarios for Airbnb and a return to the idea of SE. They forecast changes in the breakdown of hosts (scenario 4). First of all, they expect a decline/outflow of capitalist hosts, i.e., those who to date have obtained the highest profits from renting, and an increase in the share of befriender hosts and ethicist hosts [49] who hold the platform’s original ethos more dear, i.e., sharing housing space with ordinary people [46,49,82]. Some point out that the uncertainty on the travel rental market may also bring huge losses to those hosts who manage multiple accommodations (enterprise-like hosts) or significantly reduce their profits. The expected consequence is that their number of actives rentals will fall, they will withdraw from the market, or that they will even go bankrupt [66].

There is fairly widespread pessimism that tourist rental via the Airbnb platform will not return to pre-pandemic levels (scenario 1) [46,49,80,83]. Bresciani et al. (2021) predict that cities (scenario 5), and above all their previously heavily exploited, crowded centres (scenario 6), will be less attractive to tourists than before the pandemic. An increase in demand for accommodation in less crowded areas is forecasted [41,46,72,80], e.g., in the outskirts of cities (scenario 6), in mountains, in the countryside, etc. In turn, optimistic scenarios include coronavirus presenting an opportunity for changes in how not only the platform, but tourism as a whole function [23,46,64,66,67,87,88,89]. The pandemic may, above all, provide an opportunity to transform the tourism industry in a more sustainable direction [46,64,66]. In many cities, attempts are being made to reorganise tourism to function in a way that is compatible with other forms of activity [87]. As noted by Gerwe (2021), this is a unique time for regulators and local authorities to implement better practices and introduce rules and standards (see also [90]).

### 2.3. Airbnb and Tourist STR in Poland

The development of SE in the area of homesharing in Poland is quite well described for the period before the pandemic. The general outline of the Airbnb phenomenon was presented by, among others, Pawlusiński (2017) and Pawlicz (2019) and in the system of tourist metropolises and cities: Kowalczyk-Anioł, Pawlusiński (2018). A few studies on individual cities were written, mainly on Warsaw [55,91,92] and Kraków [93]. Part of the discussion was devoted to the impact of Airbnb’s activities on various areas of city functioning (e.g., [21,22,94,95,96], housing [56,91], and the rental housing market [97]. The purpose of and/or possibilities for limiting STR in Poland were also discussed [26].

The latest works considering the pandemic in analyses of the rental housing market refer indirectly to STR in Warsaw [56] and Kraków [97]. A broader view is brought by the work of Adamiak (2022), who recently showed general changes in Poland in tourist flats and registered accommodation facilities. However, there is no study to date that has investigated how the landscape of STRs, i.e., the volume and breakdown structure of the offer, changed in Polish cities during the pandemic. The remainder of this analysis will be devoted to this issue.

**Table 1:** Main possible scenarios for COVID-19-induced changes in Airbnb (STRs) featured in the literature.

	Scenario of Change	Author(s) (Date of Publication)
S1	Airbnb’s active rentals volume and popularity drop	Bugalski (2020) [83]; Dolnicar, Zare (2020) [49]; Polisetty, Kurian (2021) [80]; Gerwe (2021) [46]
S2	Transition/return to long-term rental	Boros, Dudás, Kovalcsik, (2020) [66]; Bugalski (2020) [83]; Celata, Romano, (2020) [87]; Dolnicar, Zare, (2020) [49]; Farmaki et al. (2020) [75]; Rubino, Coscia, Curto (2020) [84]; Gyódi (2021) [64]; Yiu, Cheung (2021) [74]
S3	Decrease in rental offers of flats and shared rooms; growth in entire apartments	Hu, Lee (2020) [78]; Lim et al. (2020) [30]; Bresciani et al. (2021) [47]; Benítez-Aurioles (2021) [70]
S4	Changes in host breakdown—a decline in capitalist hosts	Dolnicar, Zare (2020) [49]; Boros, Dudás, Kovalcsik (2020) [66]
S5	Fall in demand for offers in cities	Bresciani et al. (2021) [47]; Gerwe (2021) [46]; Tomal, Helbich (2022) [97]
S6	Fall in demand for offers in city centres; turn towards peripheral districts	Rubino, Coscia, Curto (2020) [84]; Bresciani et al. (2021) [47]; Boros, Kovalcsik (2021) [72]; Liang et al. (2021) [41]; Gerwe (2021) [46]; Zare, Dolnicar (2020) [49]; Polisetty, Kurian (2021) [80]; Tomal, Helbich (2022) [97]

## 3. Study Area

**Figure 1:**
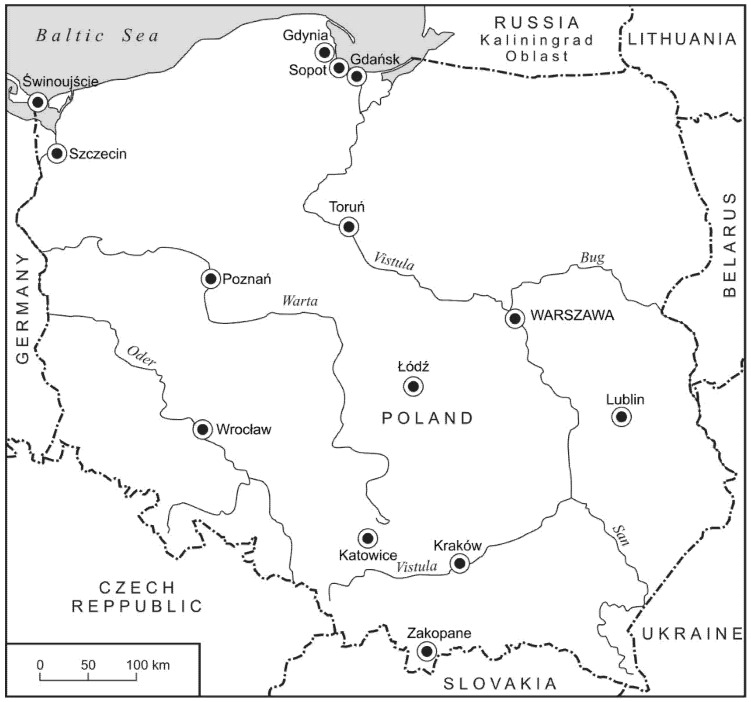
Map of Poland with the locations of the cities studied.

Fourteen cities with the largest number of active rentals in the third quarter of 2019 in various parts of Poland are analysed (Figure 1). The list of cities is based on research by Kowalczyk-Anioł, Pawlusiński (2018) and data from the AirDNA website. They differ in size, in position in the settlement hierarchy and, essentially for this study, vary in terms of tourist potential, which determines their attractiveness and thereby the development of the city’s tourist function.

Most of them are large cities, capitals of regions [Warsaw (Warszawa), Kraków, Łódź, Poznań, Wrocław, Gdańsk, Szczecin, Lublin, Katowice, Toruń], but also small cities located in areas attractive to nature.

The largest of the analysed cities is the nation’s capital, Warsaw [98]. The city rebuilt from ruins after WWII with the Old Town entered on the UNESCO World Heritage List. It has very good transport accessibility, great tourist potential, an extensive accommodation base and a very large amount of visitors [98].

The second largest is Kraków, a city with a historical heritage that attracts many tourists (over 14 million in the record year of 2019, of which over three million were foreign tourists [99]). Kraków is the second largest city in Poland, and is located in the south of the country (Figure 1). Kraków’s historic centre was entered on the first List of the UNESCO World Cultural and Natural Heritage in 1978 [93].

The next city is Łódź, one of the fastest-shrinking cities in Poland. Known as the Manchester of Poland, it was once devoted almost exclusively to the textile industry. Today, it is a post-industrial city whose tourist potential is based on the revitalisation of post-industrial complexes and successful examples of succession from industrial to service functions (by international award-winning architects) [100].

Poznań is a multifunctional city centre, an historic city with many monuments dating back to the early Middle Ages; an important academic centre with a rich international exhibition and fair tradition that attracts business tourism. A city with good transport accessibility, a multifunctional centre with an important settlement position in the country, exceeding the administrative rank of the capital of the region [101].

Wrocław, similarly to Poznań, is a centre of supra-regional importance. The city is an important academic centre in Poland and a venue for numerous cultural events that attract tourists. It is of great historical value, with the Centennial Hall being a UNESCO World Heritage site [102].

Toruń is the birthplace of Nicolaus Copernicus and is among Poland’s cities best-endowed in monuments, with unique gothic buildings and an Old Town that is a UNESCO World Heritage site [103].

Katowice is the centre of the mining industry, the capital of Poland’s coal basin. It is currently a post-industrial city and rapidly growing economic centre. With a well-developed cultural scene (it is the seat of the Polish National Radio Symphony Orchestra) it also hosts a trade fair that attracts business tourism. It is a venue for numerous national music festivals [104].

Gdańsk is the capital of an agglomeration of over one million inhabitants. Together with Sopot and Gdynia, it forms the Tri-City—a complex of three cities on the Baltic coast. One of the oldest cities in Poland, it was a Hanseatic city and has more than 1000 years of tradition and numerous Gothic monuments. The city has very good transport links and a strong cultural scene [105]. The smaller in Tri-city, city of Gdynia is young, having been established in the 1920s. Its attractive now mainly lies in its proximity of Gdańsk, its seaside location and its many related maritime attractions (museums, monuments and events) [106].

Szczecin is a city located near the Polish-German border on the border of the Oder River (120 km away from Berlin). It is an important seaport and the centre of the former shipbuilding industry, and now the yacht industry. Once a Hanseatic city with a large number of gothic monuments and a unique starry street layout often compared with Paris (designed by the same urban-planner Baron G.E. Haussmann) [107].

Lublin is a historic city, until the 18th century an important administrative, commercial and cultural centre in this part of Europe. A city attracts tourists thanks to its fascinating history and architecture where the cultures of the East and West Europe meet. Currently, an important academic and cultural centre in eastern Poland [108].

The last three cities are much smaller than the others and are characterised primarily by their attractive locations and extensive accommodation bases (Table 2). They include Sopot, the smallest city in the Tri-City, which is attractively situated on the Baltic coast and was established as a German health resort. Currently, its numerous pubs, restaurants and clubs have earned it a reputation as the entertainment centre of the Tri-City. Sporting the longest wooden pier in Europe (511 m) and a large number of tourists, it is often also referred to as the summer capital of Poland [109].

The second city in this group is the slightly larger Świnoujście, also located on the Baltic coast. A seaside tourism centre, it attracts many seasonal tourists, mainly due to its location and status as a spa. Świnoujście port is closely connected with Szczecin and has ferry connections to Sweden and Denmark [110].

The last and smallest city in this group is Zakopane. Nestled close to the Tatra Mountains, it is known as the winter capital of Poland. In winter it is a winter sports resort, while its summer pull is its mountain location and nature, mountain hiking and local highlander folk culture (architecture, music, cuisine, highland traditions, etc.) [111].

## 4. Materials and Methods

As commercial companies, STR platforms do not publish or share data on their activities, which is a fundamental obstacle to systematic research and analysis of this form of accommodation provision. Therefore, the article uses data from the AirDNA service. AirDNA is an enterprise that provides data on short-term rentals and prepares related detailed analyses for hosts, investors and companies [112]. It is the most popular commercial data provider on the short-term rental market and is based on information obtained from Airbnb and Vrbo (formerly HomeAway). According to Adamiak (2022: 14) “on the basis of automated tracking of offers on platforms (web-scraping) and data obtained from partners—mainly suppliers of software supporting rental—the platform publishes fairly accurate information about the number and characteristics of available offers and the number of days on which they were booked” (see also [15,113]). Although some (see [94]) suggest that author-made web scraping is the best method for STR analysis, many more hold that AirDNA is the best source available for research into the Airbnb (STR) market (e.g., [15,113]). With this in mind, and the fact that AirDNA is currently reporting (with the possibility of viewing earlier data) on two SE platforms, i.e., Airbnb and HomeAway (now Vrbo), AirDNA was considered an appropriate and sufficient source of data for this article.

The desk research method was used in the study. The study consisted in an analysis of data obtained from the website www.airdna.co for January 2019 to January 2022 for 14 Polish cities that, according to AirDNA, constituted the largest number of active rentals. They were Kraków, Warsaw, Gdańsk, Wrocław, Sopot, Zakopane, Gdynia, Poznań, Szczecin, Łódź, Katowice, Toruń, Lublin and Świnoujście. The analysis included information on the number of active rentals in individual cities from the first quarter of 2019 (Q1 2019) onwards and data on the number and breakdown of active rentals, including entire home rentals and private rooms. The values of these variables and their changes over time were analysed. To analyse the dynamics of changes in the number and breakdown of facilities offered via Airbnb and to determine the direction of these changes, indices were used that show relative increases/decreases in the studied variable values in a research period. The study employed both fixed-base indices that assume Q1 2019 as the starting date for the analysis and chain indices that show changes in the individual sub-periods (quarters) under study. The beginning of the COVID-19 pandemic in Poland—officially 4 March 2020, when the first case of the disease was diagnosed—was distinguished in the analysis as an important time point [114].

The analysis included a pandemic calendar, because in terms of the scope of restrictions introduced by the authorities, it was not a homogeneous period. The first restrictions were introduced on 12 March 2020 (e.g., cancellation of mass events, closure of schools, universities, suspension of cultural institutions, closure of borders, etc.). March 2020 was the month when new restrictions were announced and introduced every few days—restrictions on business activities (e.g., closing shopping centres, DIY stores, restaurants, hotels), a prohibition on visiting green areas (parks, forests, beaches), the introduction of hours dedicated for seniors to go shopping, etc. [115]. On 23 March a state of epidemic was declared, which resulted in the first lockdown restricting movement and banning assembly in groups. At the end of April, an initial lifting of restrictions was announced that lasted until early June [115,116]. In the autumn of 2020, the second wave of the pandemic began. In October, gradual restrictions were announced and in early November a new lockdown was introduced [115]. In January and February 2021, restrictions were gradually lifted, but in March a third pandemic wave was declared and another lockdown introduced. Restrictions began to be lifted before the summer months. To revive tourism and facilitate international European travel, the European Union introduced the EU Digital Covid Certificate for the fully vaccinated from 1 July 2021, which facilitated travel between EU countries and was also intended to contribute to slowly rebuilding the tourism sector [117]. In Poland, the fourth wave lasted from October to December of 2021. The fifth wave came to Poland in January 2022, with record case numbers caused by the more infectious Omicron variant. Numbers of infections, and thus introductions of numerous restrictions and lockdowns, were greatest in the autumn and winter months throughout the study period.

## 5. Results

The most important variable used to determine the dynamics of the Airbnb platform’s development—and thus how the pandemic affected its functioning—is the number of active rentals. Active rentals “are those that had at least one reserved or available day in the last month” [118].

**Figure 2:**
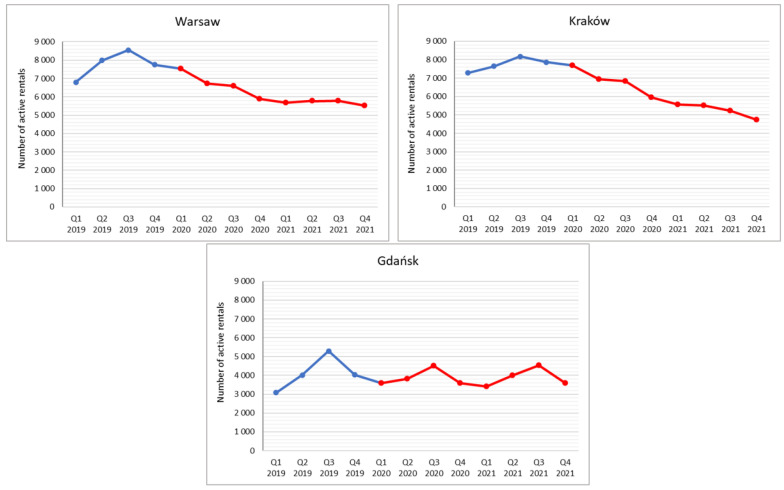
Number of active rentals by quarter (Q) (in cities with more than 3000 rentals).

**Figure 3:**
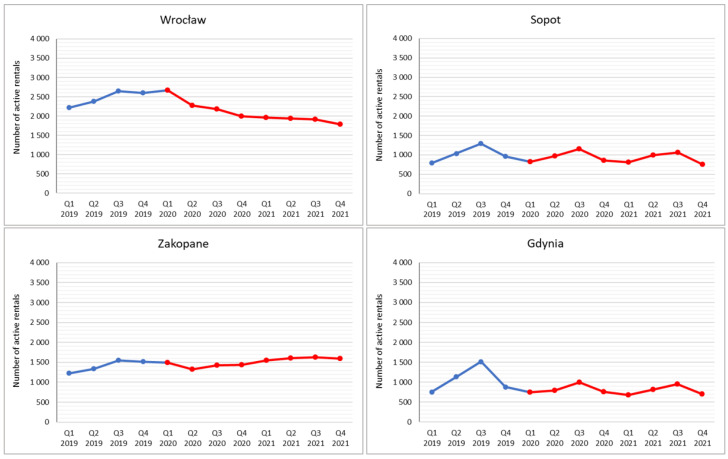

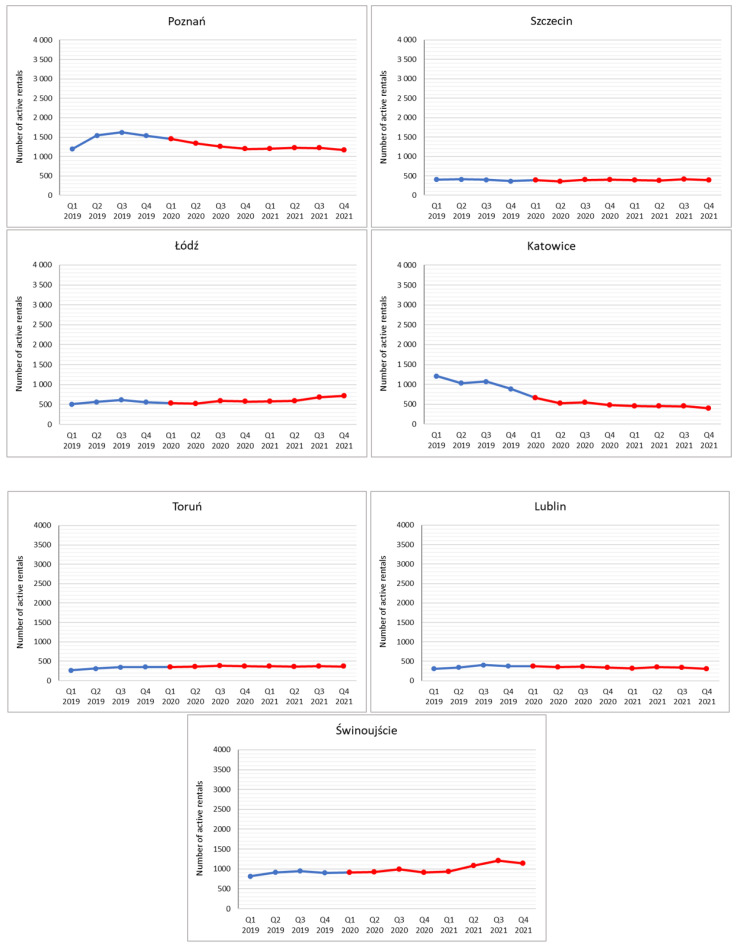
Number of active rentals by quarter (Q) (in cities with the number of rentals below 3000).

The analysis showed significant differences in active rental numbers between cities over the study period, which results how the cities differ in character. Taking into account the data for Q3 2019 (i.e., the STR platform’s peak popularity in Poland), the largest number of active rentals was over 8000 each in Warsaw and Kraków, and slightly lower (but also high relative to population) in Gdańsk at over 5000, while in some cities it did not exceed a few hundred (Figure 2 and Figure 3).

The analysis of active rentals dynamics distinguished two periods during the three study years for all major Polish cities (except Łódź), i.e., Warsaw, Kraków, Wrocław, Poznań, Katowice, Lublin and the Tri-City (Gdańsk, Gdynia, Sopot). The first is a period of growth that lasted until Q3 2019, when platform achieved peak usage, while the second is a period of decline that was then consolidated or even aggravated during the pandemic. The peak of development recorded in Q3 2019 had two determinants. The first was the rather slow but steady increase in popularity of this form of rental in Poland up to 2019 (see [52]) until saturation was achieved, and the second was the seasonality of the phenomenon, because the data for Q3 express the average of the three summer months. The decrease, meanwhile, resulted from the introduction of pandemic restrictions from the end of Q1 2020, including full or partial lockdowns, which prevented free travel and accommodation [119]. The second reason, partly independent of the restrictions, which only apply to registered accommodation establishments, was that tourists limited city breaks due to fears of crowded city centres, where Airbnb’s major-city active rentals are traditionally concentrated. This was especially true of foreign tourists, who constitute an important proportion of city visitors to major cities. Apart from the aforementioned tourist preferences and concerns, the situation can also be associated with the much reduced (by the pandemic) network of air connections [120] (especially low-cost airlines) and the constant changes in rules governing international travel restrictions [121]. Interestingly, not even periods of looser restrictions in the summer months of 2020 reversed the downward trend. Nor too did the largest seasonal increases in active rentals recorded in the summer months in the Gdańsk, Gdynia and Sopot (Tri-City) seaside area reverse this trend, merely moderating its trajectory. It is worth noting that the greatest decrease in rental properties was in Kraków, which had had the most active rentals in the country in the initial study period; this made Warsaw the new leader in terms of active rental numbers in Poland as of Q1 2021.

The situation was different in smaller cities in areas of natural beauty (mountainous Zakopane and seaside Świnoujście), with a constant increase in active rentals throughout the analysed period, regardless of the pandemic. This increase was probably also caused by pandemic restrictions that limited Poles’ capacity to travel abroad and the aforementioned restriction on city breaks that turned their attention to smaller cities and places in areas of natural beauty, and in this case even to health resort areas. This may also have been influenced by the actions of the Polish authorities and the introduction of a travel voucher that could be redeemed against payment for various tourist services, including accommodation in appropriately registered domestic establishments. A one-time voucher was made available in the amount of PLN 500 per child under 18 (or PLN 1000 for a child with a certified disability). The campaign was launched on 1 August 2020 and is expected to last until the end of September 2022. Importantly, entitlement to a voucher was not based on level of income. The government estimated that approximately 6.5 million Polish children would be able to take advantage of it. The voucher was designed to support not only Polish families but also the tourism industry in the difficult pandemic period [122]. According to data from Poland’s social insurance institution, ZUS, published in a report by the Polish Tourist Organisation (POT) and the Ministry of Development and Technology on 30 September 2021, 7.3 million vouchers had been generated. By the end of August 2021, 2.6 million payments had already been made using the Polish Tourist Voucher, bringing the tourism industry a total of over PLN 1.7 billion [123].

It is interesting that, during the pandemic, there were also increased numbers of active rentals in a completely different type of city centre than those mentioned above. This was the country’s third-largest city by population—post-industrial Łódź, where the small number of active rentals for a large city (only 600 at the peak recorded in Q3 2019) not only did not shrink during the pandemic, but even increased. The second such city is a bit smaller, a provincial capital, the historical city of Toruń with its medieval Old Town (UNESCO WHS). These two cases appear to be explained by Airbnb having previously been poorly developed relative to their populations and tourist potentials. And although the typical tourist profile in internationally renowned, historic Toruń is completely different from that of post-industrial, niche tourist Łódź, the pandemic turned out to have no (overall negative) impact on the development of Airbnb in either of these centres.

The last of the examined cities, Szczecin, does not fit into any of the above-mentioned groups: the pandemic had no impact on the size of the STR offer, which remained almost unchanged throughout the entire study period.

**Figure 4:**
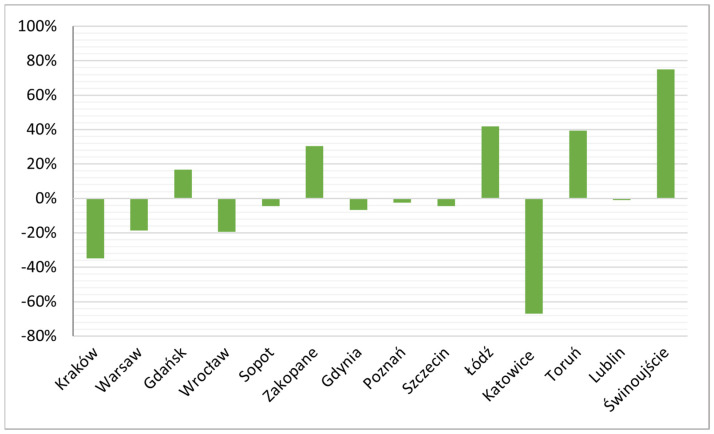
Index of dynamics of changes from Q1 2019 to Q4 2021. (Q means quarter).

A closer analysis of the dynamics of the phenomenon, expressed in indices of changes, showed that for the entire research period (Q1 2019 to Q4 2021), the largest decreases were recorded in Katowice (67%), Kraków (35%), Wrocław and Warsaw (19% each). Very slight decreases of just a few percent were recorded in Gdynia (7%), Sopot and Szczecin (4% each). Interestingly, in Gdynia and Sopot’s neighbour, Gdańsk, there was an increase of 17% (Figure 4), although, as already stated above, the number of active rentals in this city has decreased since the 2019 peak. Of the examined cities, the largest increases were recorded in Świnoujście (75%), Łódź (42%), Toruń (39%) and Zakopane (31%). In Lublin and Poznań, the number of active rentals barely changed in the analysed period.

**Figure 5:**
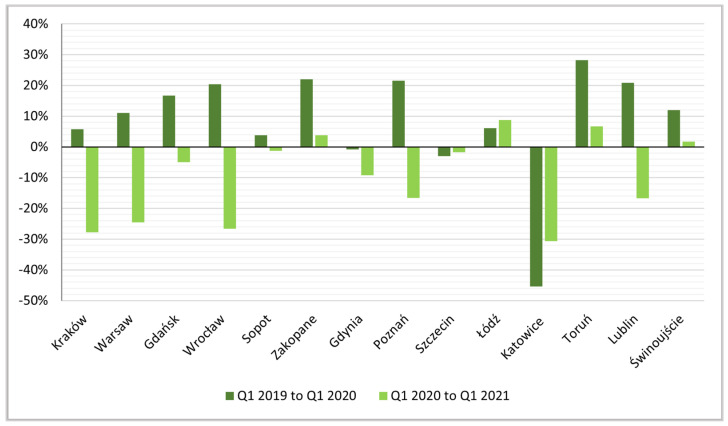
Indices of dynamics of changes, Q1 2019 to Q1 2020 and Q1 2020 to Q1 2021.

A closer analysis accounting for seasonality by showing dynamics between successive, analogous quarters of the analysed years, shows the pandemic had a strong negative impact on active rental numbers. In almost all of the surveyed urban centres (except Gdynia, Szczecin and Katowice) from Q1 2019 to Q1 2020 there was an increase of several to almost thirty percent (Toruń). In the next analysed period, Q1 2020 to Q1 2021, decreases were recorded due to the pandemic-induced collapse in urban tourism (Figure 5) The exceptions were Zakopane, Łódź, Toruń and Świnoujście.

**Figure 6:**
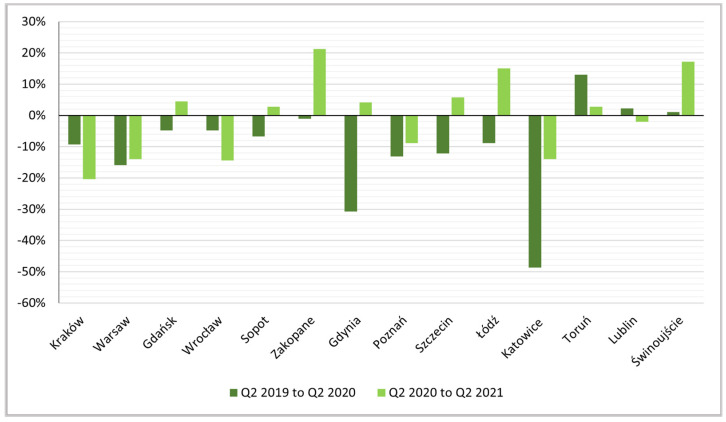
Indices of dynamics of changes, Q2 2019 to Q2 2020 and Q2 2020 to Q2 2021.

**Figure 7:**
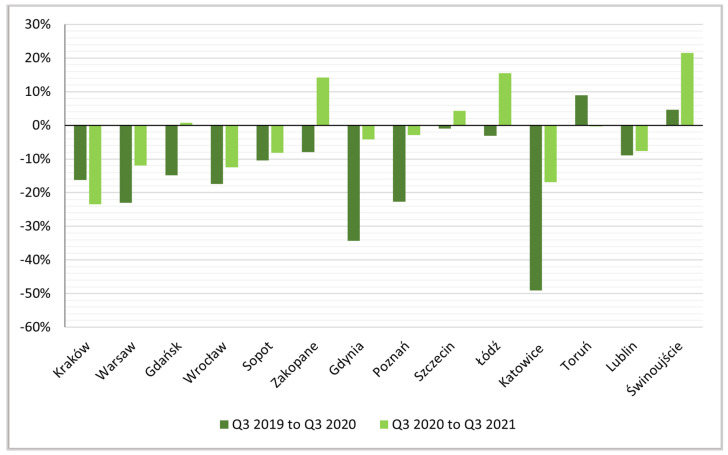
Indices of dynamics of changes, Q3 2019 to Q3 2020 and Q3 2020 to Q3 2021.

**Figure 8:**
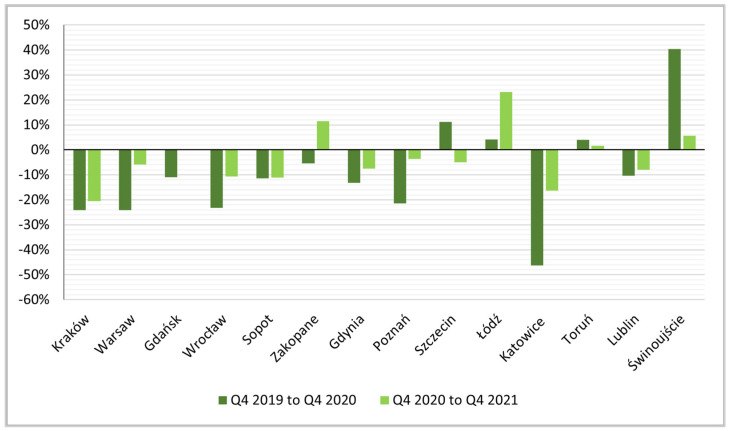
Indices of dynamics of changes, Q4 2019 to Q4 2020 and Q4 2020 to Q4 2021.

The negative impact of the pandemic on the number of active rentals is also shown by the dynamics indices for the other quarters (Figure 6, Figure 7 and Figure 8).

The data show that, although there were slight increases in the period between the following quarters (mainly in the second analyzed sub-period) in some cities, in successive quarters of 2019 (Q2, Q3 and Q4) and the same quarters of 2020 (i.e., in the subperiod covering the first quarters of the pandemic), there were more or less deep losses in the number of offers almost everywhere. The exceptions are nature-based tourism and health destinations—seaside Świnoujście on the Polish–German border, which gained over the entire study period (not only in the first year of the pandemic but also in the second) and the mountain tourism destination of Zakopane. This was related both to the revival of domestic tourism in traditional nature-based regions that avoided major cities and restrictions that restricted access to “official” accommodation offerings (hotels, hostels, etc.). During the pandemic (2020), despite the temporary suspension of services, the region in which Świnoujście is located attracted the largest number of health tourists in Poland [124]. Świnoujście is distinguished by a steadily emerging base of high-quality apartments, in both the seaside and spa zones [125], which contributed to the observed increase in STR offer. Moreover, one can expect the increase in STR saturation to continue at this destination, given that it has a highly developed tourist function (see Table 2) and one of the highest shares of foreign tourists (mainly German) in the breakdown of visitors before the pandemic in Poland.

The forecast scenarios of pandemic impact on STR platform growth also include active rentals broken down between entire home and private room rentals.

**Figure 9:**
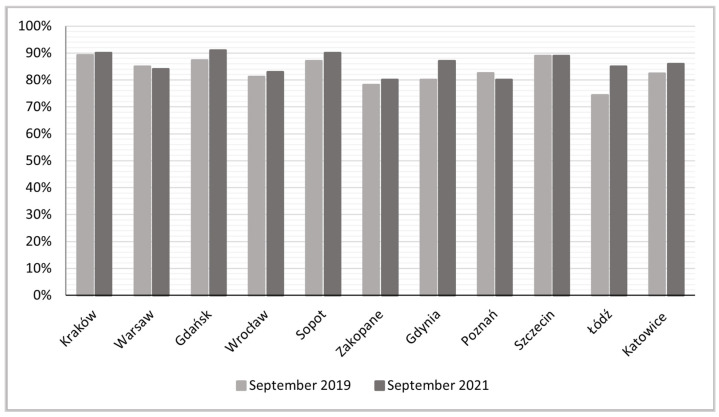
Share of entire home rentals in the total Airbnb and Vrbo offer.

A closer look at the shares of entire home rentals from September 2019 to September 2021 reveals that the previously high share values of this type of accommodation (exceeding 80% in most cities) increased or remained unchanged in almost all cities (Figure 9). Only in two, Warsaw and Poznań, was a very slight decrease recorded.

**Figure 10:**
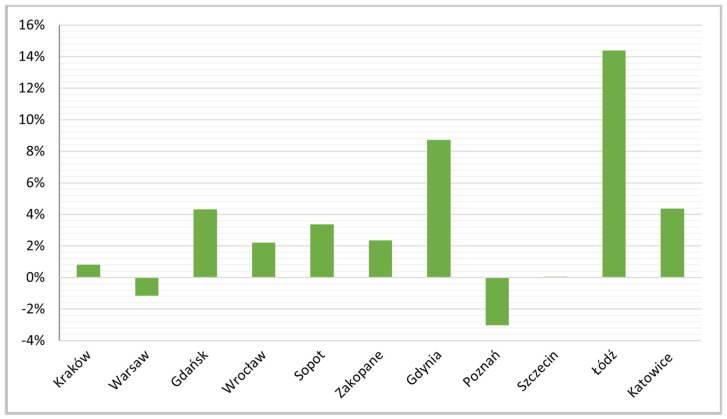
Growth dynamics indices for entire home rentals, September 2019 to September 2021.

The proportional increases in entire home rentals in this period were highest in Łódź (14%) and Gdynia (8%), and with it the entire Tri-City (Gdańsk and Sopot) and Katowice (the post-industrial city) (Figure 10). The relatively small increase in this form of accommodation as a share of the breakdown of total Airbnb active rentals is probably due to its high starting level, but the COVID-19 pandemic certainly contributed to consolidating this trend. Similar conclusions have been reached by other authors (e.g., [47,70]).

**Figure 11:**
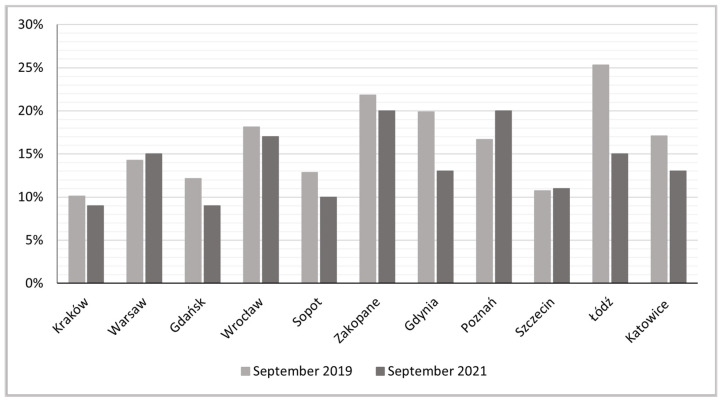
Shares of private rooms in total Airbnb and Vrbo active rentals.

**Figure 12:**
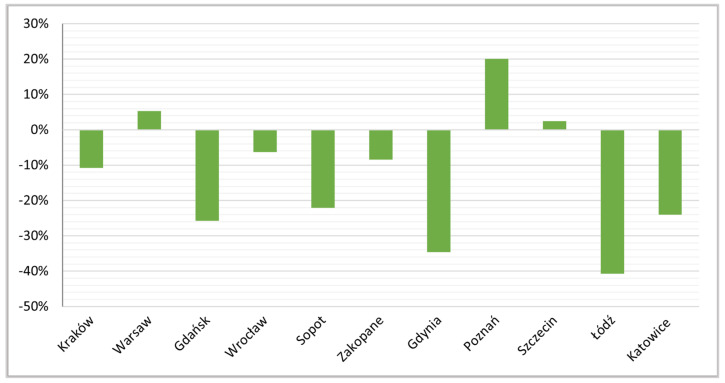
Indices of dynamics of changes in proportion of private rooms, Sept 2019 to Sept 2021.

In September 2019, in the examined cities, private rooms represented from 10% (Kraków) to over 25% (Łódź) of active rentals, but in the study period most of these percentages dropped in most of the cities, except Warsaw, Poznań and Szczecin (Figure 11). The largest decreases (of over 40%) were recorded in Łódź, where their initial level had been highest, and in the Tri-City—mainly in Gdynia (by over 30%) (Figure 12).

## 6. Discussion and Conclusions

The unprecedented pandemic-induced changes in most social and economic spheres of life in cities were global [126,127,128,129], and the tourism sector was one of the very heavily affected economic sectors [64,90,130]. Particularly large changes were thus noted in tourist cities—especially those with a highly international tourist offer that prior to the pandemic had experienced a growth and proliferation of STRs via global platforms, especially Airbnb [61,64]. The outbreak of the pandemic initiated a new phase in the operation of STRs in cities and in related research. The article therefore addresses both issues. First, scenarios of potential pandemic-induced changes for Airbnb/STRs in the literature are presented (see Table 1), and then a case study of cities in Poland shows how the number and breakdown of STRs (Airbnb and Vrbo) changed during the pandemic.

Despite the fairly widespread belief that the pandemic would cause a drop in the volume and popularity of mediated homesharing platforms (scenario S1 of Table 1), especially in cities (scenario S5 of Table 1), the conducted research shows contrary results for the pool of cities studied in Poland. The surveyed centres include some that confirm these predictions (Warsaw, Kraków, Wrocław, Poznań, Katowice, Lublin) and some that show the converse course of the phenomenon (a steady increase in active rentals in Łódź, Świnoujście, Zakopane, Toruń). The first group comprises major cities, especially Warsaw, Kraków, Wrocław and Poznań, where the number of active rentals decreased quarter on quarter through the pandemic. These are well-connected major urban centres, the most popular urban tourism destinations, and popular city-break destinations in Poland. Warsaw and Kraków also had the largest volumes of active rentals on Airbnb and Vrbo in Poland.

The results presented in the article can also be partially explained by the conclusion of the authors of the Fortech Consulting report [57] that on the eve of the outbreak of the pandemic, i.e., in September 2019, there were symptoms of market saturation with tourist apartments in five major tourist destinations—Warsaw, Kraków, Gdańsk, Sopot and Wrocław. At that time, there was a drop in average occupancy and daily rental rates, and some apartments began to migrate from the short-term model back onto the long-term rentals market [57]. The results confirm a gradual decrease in the number of STR apartments in these centres in 2020 and 2021. At the same time, in the Tri-City (Gdańsk, Gdynia, Sopot) there were periodic increases (in third quarter of 2020 and third quarter of 2021) related to both the traditional seasonal nature of tourist traffic in these destinations and the easing of COVID mobility restrictions during the holiday season [119].

An additional interesting commentary on the research is provided by Tomal and Helbich’s (2022) analysis of the Kraków housing market. It shows that the COVID-19 pandemic saw rents fall significantly all across Kraków, and proximity to the city centre also became a significantly weaker determinant of rent level (see also [97]). The real situation is explained by Tomal and Helbich (2022) by the influx of short-term rented apartments, mainly from Airbnb, onto the long-term rental market. A similar mechanism was identified in Warsaw by Trojanek et al. (2021). It can therefore be assumed that, for Kraków and Warsaw (tourist destinations already saturated with STRs), scenario S2 of Table 1 (that “holiday apartments/homes would be returned to the regular rental market”) came to fruition. However, similarly to Kadi et al. (2020), we tend towards the assumption that a current shift to the regular rental market is likely, but that the medium- and long-term development is uncertain. Moreover, preliminary Airbnb observations for March and April of 2022 have shown that the war in Ukraine has spurred a jump in rental rates in all major Polish cities, including for STRs such as Airbnb. This new phenomenon, unprecedented on the STR market, certainly requires further research.

The second group was of cities where, contrary to the scenarios in the literature, there was an increase in the volume of active rentals. These include the mountain town of Zakopane and coastal Świnoujście (both located in areas of natural beauty), post-industrial Łódź and the slightly smaller, but very attractive tourist destination of Toruń. While the increases in Zakopane and Świnoujście can be explained by the increase in demand for domestic travel and perhaps the government’s tourist voucher incentive, the growing share of active rentals in Łódź and Toruń seems to be due to the low initial level of STRs in these cities and its continued upward trajectory despite the pandemic.

Therefore, comparing the obtained results to the research objective set out in the introduction, which was to determine the size and direction of changes in the STR offer in Polish cities, it can be concluded that these changes differ in pace and direction among cities. However, the analysis revealed certain regularities in these changes related to their size, the pre-pandemic level of saturation of the accommodation offer (including the STR offer) and location (in particular with regard to areas of natural beauty), and, based on this, revealed two main groups among the cities discussed above. The first regularity involved a decrease in the STR offer during the pandemic and related to the largest cities with the most developed accommodation offer, and the second involved an increase in the STR offer and related to cities in areas of natural beauty and those with a relatively small accommodation base and low pre-pandemic saturation of STR offer.

Summing up, it is worth noting, after Adamiak (2022), that the pandemic-induced decrease in numbers of tourist trips in Poland was less noticeable in tourist apartments (STRs—many of which, as already mentioned, are “invisible” to official statistics) than in the registered accommodation base (such as hotels, hostels etc.).

Another scenario that can be verified on the example of Polish cities is the decline in active rentals for apartments and shared rooms, and thus the proportional increase of entire apartments among active STRs (S3). This was confirmed (although the changes were small) because, as Kowalczyk-Anioł and Pawlusiński (2018) showed, the pre-pandemic stage of development in Polish cities was typified by a high and constantly growing share of entire homes [14], attesting to the progressive financialisation of the tourist STR market [95]. A change in the data presentation method (i.e., the deletion of information about the participation of “multi-hosts” among STR hosts) makes it impossible to directly analyse the scenario forecasting an outflow of capitalist hosts from the tourist STR market (S4). However, the analysis of the breakdown of hosts presented in the article did not show a significant change during the pandemic; it can thus be assumed that the participation of so-called befriender hosts and ethicist hosts [49], who share their living space with tourists (shared rooms or shared apartments) remains insignificant in Polish cities. The remaining scenarios (S5 and S6) refer to the demand for tourist STRs, so cannot be interpreted in the light of the supply research presented in this article. They require in-depth research, but, as Braje et al. (2021) indicate, the pandemic barely changed most of its existing users’ desire to use Airbnb again in the future. Jang et al. (2021) also showed that business tourists (who perceived the threat of COVID-19 as low) are more willing to consume urban Airbnb listings than are leisure tourists.

Rutkowska-Gurak and Adamska (2019), in analysing the consequences of SE development for cities before the pandemic, emphasised that they are quickly changing over time, because they change as the strategies of the corporations themselves change, and as the activities of other stakeholders adapt. The pandemic confirmed these observations and added new contexts to them. First of all, it highlighted the importance of geographical context—different countries, regions and cities were affected differently by the waves of the pandemic and adopted various policies on tourism and sharing economy in tourism (including STRs) that changed over time. In this article, we have shown some insights into part of the CEE. Moreover, in some cities and regions, homesharing platform users were only a small proportion of non-resident city users (e.g., those engaged in workations [65,131]) and of the first tourists between waves of lockdowns (especially business tourists [132]) and/or when cities reopened to tourism. Therefore, when planning further studies on STR consequences of the pandemic, it is also worth paraphrasing Ioannides’ question (2016): Do activities such as Airbnb (tourist STR) strengthen the opportunity for bottom-up resilience to pandemic in the tourism city? The results presented in this article suggest that, for the largest cities in Poland (Warsaw and Kraków), STR market resources not used by tourists may have unexpectedly contributed to housing resilience (at least to greater availability of housing) during the pandemic by being put back on the traditional rental market, thereby increasing supply and lowering prices. These are new issues that are also associated with the difficult-to-assess nature of the sharing economy in tourism.

The analyses presented in the article have limitations that are mainly related to the nature of the available data, as emphasised in the methodology section. In subsequent studies, it will also be worth examining and comparing the mechanisms by which the pandemic affected traditional accommodation facilities. It will also be worth exploring more deeply and monitoring the changes in the relationship between tourist homesharing and conventional rental, as emphasised earlier. At the same time, the analyses and conclusions presented may be useful both for local tourism stakeholders (including Destination Marketing Organizations) and for the city authorities developing optimal and effective post-pandemic tourism management policies in the city and (regulation of) short-term tourism rentals.

The article documents the changes in STRs in Poland before the escalation of armed conflict between Russia and Ukraine (24 February 2022). This conflict is becoming an important factor in the development of STRs in Central and Eastern European cities, and for the analysis of this phenomenon in the studied region. As this article shows, STRs (but also, more broadly, the sharing economy in tourism) in cities are in continual flux, so further studies are necessary—both theoretical and empirical.

## Figures and Tables

**Table 2:** The analyzed cities ranked according to the number of population.

	Population (2020)	Area (km^2^) (2021)	Number of Active Rentals in the End of Q2 2021 * Per 1000 Inhabitants	Number of Tourist Beds in the End of Q2 2021 * Per 1000 Inhabitants
Warsaw	1,794,166	517.2	3.2	20.9
Kraków	779,966	326.9	7.1	38.5
Łódź	672,185	293.3	0.9	9.8
Wrocław	641,928	292.8	3.0	21.3
Poznań	532,048	261.9	2.3	16.6
Gdańsk	470,805	262	8.5	41.4
Szczecin	398,255	300.6	1.0	18.8
Lublin	338,586	147.5	1.0	14.4
Katowice	290,553	164.6	1.6	16.6
Gdynia	244,969	135.1	3.3	14.0
Toruń	198,613	115.7	1.8	20.3
Świnoujście	40,948	202.1	26.5	257.7
Sopot	35,286	17.3	28.1	196.8
Zakopane	26,846	84.3	59.8	540.9

* Q—means quarter.

## Data Availability

The published statistical data come from the AirDNA website. Not all of them are still available on the website, due to the fact that AirDNA regularly updates the data and the free version of the website does not have access to the archive data.
